# Medical Education

**Published:** 1891-12

**Authors:** 


*With this special issue of* THE JOURNAL, *the proprietors have concluded to reduce the subscription price to two dollars per annum, in advance. We have been induced to make this reduction for several considerations, one of which is, the matter of convenience to our subscribers who choose to send their money by mail.* THE JOURNAL *is in better condition to-day than ever before, and it is the wish, and will be the effort, of the editors and proprietors to give our readers the best journal for the price that is published in the South.*

### MEDICAL EDUCATION.

(The following article is an abstract of an able and timely paper read at the late meeting of the Virginia Medical Society by our friend, Dr. Chas. M. Blackford, of Lynchburg. We only regret that we cannot print the paper in full.—ED.)

The facilities for instruction, which the medical student of to-day enjoys, are superior in many respects to those of his predecessor of the last generation. The reduction in price of the higher literary learning enables him to receive a better preliminary training than was formerly available, and the liberal endowments of medical schools and hospitals give laboratory and clinical opportunities that before had to be sought in the great universities of the Old World.

But as the strength of a chain is but the strength of its weakest link, so the merits or demerits of our American system lie not so much in the opportunities which the student may embrace, as in the proficiency required of him before he is declared by his college to be fitted to practice. I have before me as I write the annual announcements of twelve American medical schools, and of these one requires twenty-seven months' study for its degree, two require twenty-one months, one requires twenty, two require eighteen, one requires fifteen, four require twelve and one requires ten. These all require at least two, and many three, terms, but the collegiate year in but one of these colleges exceeds seven months, and in one is but five. When the varied scientific information which is demanded of the physician to-day is considered, it seems hardly possible that a satisfactory knowledge of the science of medicine, to say nothing of the acquirement of the art of treatment, can be obtained in so short a time. In three years, a young man is expected to become a master of Anatomy, Chemistry, Physics, Physiology, Materia Medica, Hygiene, Surgery in all its branches, Medicine and Medical Jurisprudence, to say nothing of Pathology, Bacteriology and Electricity. According to published statements, the same course requires five years in England and eight in Germany, and making all due allowance for the genius of American students, the discrepancy is striking.

As a result of the ease with which diplomas may be obtained under our present system, many States, including our own, have found it necessary to establish boards of examiners whose duty it is to see that applicants for licenses are properly educated. The finding of this board in Virginia is far from reassuring to those States in which no such law exists, for, from their report for 1888, we find that 22.5 per cent. of the applicants before it have been refused license. New York has also attacked this evil, and has struck at its source by prescribing a three years'

graded course as necessary for the degree of Doctor of Medicine from any of her schools, and requiring intending matriculates to undergo a preliminary examination in arithmetic, geography, grammar, orthography, American history, English composition and the elements of natural philosophy. This examination is not conducted by the authorities of the medical schools, but by the Regents of the University of New York. In thus putting the entrance examination in the hands of those who are not interested financially in the admission or rejection of candidates, a great step in advance has been made. In addition to this provision, intended to raise the educational grade of those entering on the study of medicine, a State Board of Medical Examiners has also been established whose duty is similar to that of the Virginia Board. In North Carolina a like safeguard exists, as also in a few other States. In this way a protest has been made by some of our legislatures against the reckless way in which too many of our schools grant diplomas, but so long as there is no limit to the number of medical schools, this evil will exist, for we cannot expect a school whose existence depends on its tuition fees to commit suicide to elevate the dignity of our profession.

How then shall the standard of medical education in this country be raised? I cannot presume to dictate, but will only offer a mode that is self-evident. As the the evil is two-fold, so must the remedy be. The laity must be educated more thoroughly in an appreciation of the highly educated medical man. We laugh at the superstitions of the middle ages, and yet we have almost as many around us to-day. Instances of this can be seen everywhere. In my own experience, I have seen men of education who habitually carried potatoes in their pockets to prevent rheumatism. Among the colored population, the belief in Voodooism is wide-spread, and is not a negligable factor in the treatment of their diseases. The veil of mysticism that surround the physician and his work must be swept away, and the laity educated to an appreciation of his labors. The principles of hygiene should be taught in the public schools, and the reasons for them given so clearly as to be understood by all, for "the reason of the law is the life of the law," and unless the reason be seen, the laws of health will be but a senseless code of rules.

When we get an intelligent and appreciative body of laymen, we will find that it will no longer tolerate the half-finished product of many of our schools as at present managed. The low requirements for entrance and for graduation will have to be raised, and the American schools have it in their power to effect this renaissance of their own motion and not as the result of pressure from without.

The thought and services of the physician have been demanded by man ever since the primal curse of his race robbed him of immortality and condemned him to return to the dust of his genesis through a life of sorrow and suffering; yet thousands of years have not sufficed to separate the truths from the superstitions of pathology nor, in the minds of the public, to divorce the true physician from the quack or the conjurer.

Nor can it be said that the public has no grounds for some misgiving. Men still suffer and die, and physicians who have probed all that we have yet learned of human disease and of remedies, stand by the bed-side, helpless and hopeless. It is true that all men must die, but, as in the science of human rights, there is a remedy for every wrong, so somewhere in her great dispensary, perhaps, has Nature provided a remedy for every disease, and only waits for man to find out thoroughly what the disease is and how it is caused, that she may, in the plenitude of her mercy, banish pain and leave the physical man with no foe but the slow though sure attrition which, with advancing years, in obedience to the laws of God, wears away the strongest.

